# Functional Bi_2_O_3_/Gd_2_O_3_ Silica-Coated Structures for Improvement of Early Age and Radiation Shielding Performance of Cement Pastes

**DOI:** 10.3390/nano14020168

**Published:** 2024-01-12

**Authors:** Krzysztof Cendrowski, Karol Federowicz, Mateusz Techman, Mehdi Chougan, Ahmed M. El-Khayatt, H. A. Saudi, Tomasz Kędzierski, Ewa Mijowska, Jarosław Strzałkowski, Daniel Sibera, Mohamed Abd Elrahman, Pawel Sikora

**Affiliations:** 1 Faculty of Civil and Environmental Engineering, West Pomeranian University of Technology in Szczecin, 70-311 Szczecin, Poland; krzysztof.cendrowski@zut.edu.pl (K.C.); karol.federowicz@zut.edu.pl (K.F.); mateusz.techman@zut.edu.pl (M.T.); jaroslaw.strzalkowski@zut.edu.pl (J.S.); daniel.sibera@zut.edu.pl (D.S.); 2 Department of Civil and Environmental Engineering, Brunel University London, Uxbridge UB8 3PH, UK; mehdi.chougan2@brunel.ac.uk; 3 Department of Physics, College of Science, Imam Mohammad Ibn Saud Islamic University (IMSIU), Riyadh 11564, Saudi Arabia; aelkhayatt@gmail.com; 4 Reactor Physics Department, Nuclear Research Centre, Atomic Energy Authority, Cairo 13759, Egypt; 5 Department of Physics, Faculty of Science, Al-Azhar University, Women Branch, Nasr City 11754, Egypt; heba_saudi@hotmail.com; 6 Department of Nanomaterials Physicochemistry, Faculty of Chemical Technology and Engineering, West Pomeranian University of Technology in Szczecin, 70-310 Szczecin, Poland; tomasz.kedzierski@zut.edu.pl (T.K.); emijowska@zut.edu.pl (E.M.); 7 Structural Engineering Department, Mansoura University, Mansoura City 35516, Egypt; mohamedattia@mans.edu.eg

**Keywords:** bismuth oxide, gadolinium oxide, sol-gel, nanosilica, cement, radiation, gamma-ray, neutron, rheology, hydration

## Abstract

This study presents a new approach towards the production of sol-gel silica-coated Bi_2_O_3_/Gd_2_O_3_ cement additives towards the improvement of early mechanical performance and radiation attenuation. Two types of silica coatings, which varied in synthesis method and morphology, were used to coat Bi_2_O_3_/Gd_2_O_3_ structures and evaluated as a cement filler in Portland cement pastes. Isothermal calorimetry studies and early strength evaluations confirmed that both proposed coating types can overcome retarded cement hydration process, attributed to Bi_2_O_3_ presence, resulting in improved one day compressive strength by 300% and 251% (depending on coating method) when compared to paste containing pristine Bi_2_O_3_ and Gd_2_O_3_ particles. Moreover, depending on the type of chosen coating type, various rheological performances of cement pastes can be achieved. Thanks to the proposed combination of materials, both gamma-rays and slow neutron attenuation in cement pastes can be simultaneously improved. The introduction of silica coating resulted in an increment of the gamma-ray and neutron shielding thanks to the increased probability of radiation interaction. Along with the positive early age effects of the synthesized structures, the 28 day mechanical performance of cement pastes was not suppressed, and was found to be comparable to that of the control specimen. As an outcome, silica-coated structures can be successfully used in radiation-shielding cement-based composites, e.g. with demanding early age performances.

## 1. Introduction

Concrete is the most popular material used for radiation shielding due to low costs, global availability of its constituents, sufficient durability and its ability to form any shape required [1,2]. Moreover, the properties of cement-based composites can be easily altered by the introduction of various heavyweight fine and coarse materials [3,4]. Thanks to versatility in designing the mixture composition, concrete can be tailored to sufficiently attenuate all types of ionizing radiation, with particular emphasis on gamma-rays and neutrons [5,6]. 

To ensure adequate gamma-ray attenuation, materials with a high atomic number (Z) are required to facilitate higher interaction probability with photons. One of the common solutions is to use lead. Although lead (Pb) is a naturally occurring metal, it is extremely toxic. Therefore, various alternative material solutions are sought. To date, the highest attention has been paid to Bi_2_O_3_ [7,8,9,10] followed by TiO_2_ [11,12,13,14], Fe_2_O_3_ [15,16,17] or WO_3_ [18,19,20,21,22]. Bismuth oxide exhibits similarly high radiation attenuating properties regarding gamma radiation as lead, but is considered non-toxic. 

To effectively attenuate neutrons, materials with high neutron cross-sections are necessary [23,24]. These materials include both light and heavy elements. Heavy elements cause significant energy loss for neutrons through inelastic scattering [25]. On the other hand, elastic scattering by hydrogen (or moderator elements) results in considerable energy transfer, causing the neutron to slow down towards the thermal region [24]. A thermal neutron is a neutron that is not bound within an atomic nucleus and has an energy of around 25 meV. This energy corresponds to the most likely speed at 270 K in the Boltzmann distribution. Thermal neutrons typically have a significantly larger effective neutron absorption cross-section for a given nuclide compared to fast neutrons (which have an energy > 1 eV). Therefore, in shielding design, our goal is to slow down fast neutrons to the thermal region in order to enhance their absorption [26]. Neutron-absorbing substances such as gadolinium can then absorb these thermal neutrons. Gadolinium (^157^Gd, with a natural abundance of 15.7%) possesses the highest thermal (< 0.1 eV) neutron capture cross-section among all known stable isotopes [27]. 

While the replacement of natural aggregates with heavyweight aggregates in the concrete mix design seems relatively simple technologically, the inclusion of powdered additives (usually as cement filler) is generally linked with noticeable alterations to the cement hydration process. In recent years, there has been a visible interest in the introduction of nanosized admixtures into cement-based composites, as their performance is superior to their micro-sized counterparts [8,9,10,21]. It was already confirmed in other radiation shielding materials such as polymers [28,29], glass [30,31] and clays [32,33,34], that nanosized admixtures exhibit superior attenuating properties than their micro-sized counterparts. Primary factors attributable to this behavior are (1) surface-to-volume ratio and (2) quantum confinement effects [35]. Similarly, various comparative studies on cement-based materials have already confirmed the superiority of nanosized PbO [22], WO_3_ [22] and Bi_2_O_3_ [9,10] over coarser particles.

However, based on the polymer [36,37] and glass [38] technology experiences, in order to properly and effectively improve the radiation shielding of material it is necessary to use a combination of particles countering specific radiation sources [39]. It can be achieved either by mixing particular materials separately or by producing advanced functional fillers such as W/Gd_2_O_3_ structures [40,41] or cobalt-doped titania nanocomposites [42]. To date, the latter approach has not been reported in the literature related to concrete technology. At the same time, the incorporation of bulk mixtures of materials is available only in a few works. For instance, Soni et al. [43] combined WC and B_4_C particles in concrete, while Nikbin et al. [20] proposed the introduction of nano-Bi_2_O_3_ and nano-WO_3_ particles for targeted improvement of radiation shielding.

Although many available state-of-the-art studies evaluated the attenuating properties [9,10,20] solely, low interest was paid to the hydration, rheological and fresh performances of cement-based composites containing nano-admixtures. Knowledge related to these aspects is critical, as the amount of nano-admixtures used for radiation shielding purposes usually exceeds the optimal value for ensuring proper mechanical and durability performance of cement-based composites. It is widely agreed that, typically, the optimal content of nanoparticles lies within the range of 0.5 to 5% mass of binder [44,45,46]. However, for radiation shielding applications, the amount of a single nano-admixture frequently ranges between 3 and 10 wt% [8,10,12,13,14,17,47], therefore, higher replacement rates of a binder with nanoparticles might lead to undesirable alterations in the hydration process of cementitious systems.

For instance, both Bi_2_O_3_ micro- and nanosized particles were found to dramatically retard the hydration process, which was reflected in extremely low mechanical performance values of cement pastes [8,47]. Such performances might prevent the use of Bi_2_O_3_ nanoparticles in technologies such as repairing mortars, prefabrication technology or additive manufacturing (3D printing), where early setting times and improved early strengths are crucial. Available literature confirms that both nanosized [8,47] and microsized [48,49,50] Bi_2_O_3_ particles substantially retard the hydration process of cement, with a higher magnitude when nanoparticles are present in the cementitious system. A calorimetric study performed by Cendrowski et al. [47] reported that replacement of cement with 5 wt% and 10 wt% of Bi_2_O_3_ nanoparticles resulted in delayed occurrences of the second exothermic peak by 73% and 132% when compared to plain cementitious system with a water-to-binder (w/b) ratio equal to 0.4.

Similarly, knowledge of the performance of Gd_2_O_3_-modified cementitious systems is extremely limited to very few research works that did not deal directly with the radiation shielding performance of the material. In contrast to Bi_2_O_3_, previous studies strongly suggested that Gd_2_O_3_ particles exhibit a relatively neutral and limited effect on the fresh and hardened properties of cement-based composites [47,51].

## 2. Research Significance

To date, there has been a very limited amount of work that has reported attempts to improve both the gamma-ray and neutron shielding performance of cement-based composites by the inclusion of micro- and nanosized additives. In addition, these works were limited only to the incorporation of bulk mixtures of materials. This study aims to fill the gap in the state-of-art and propose, for the very first time, a synthesis method of functional additives for simultaneous improvement of gamma-ray and neutron attenuation in cement-based composites. The proposed structures are composed of Bi_2_O_3_ and Gd_2_O_3_ particles and SiO_2_ coating for addressing both radiation shielding performance and reducing the negative effect of the early setting of cementitious composite. To mitigate the obstacles related to the highly delayed hydration process and strength gain of a cementitious system containing Bi_2_O_3_ nanoparticles, a sol-gel coating method was introduced to produce a functional additive. The sol-gel coating is the most popular method for improving the reactivity of nanoparticles. The synthesis of thin reactive silica layers on the surface of particles enhances reactivity and incorporates nanosized admixtures in the cementitious matrix. Such technology was already applied to produce silica-coated Fe_3_O_4_ [52], TiO_2_ [53], carbon nanotubes [54] as well as steel fibers [55] for cement-based concrete applications.

In this study, two types of silica coatings were proposed. By recommending two synthesis methods, Bi_2_O_3_-Gd_2_O_3_-SiO_2_ structures are synthesized with varied silica morphology, reactivity and specific surface area. Afterward, synthesized functional additives are evaluated in standard Portland cement to assess the performance of structures in a cementitious system. Both hydration/early age performance and radiation shielding properties of cement pastes were evaluated. For comparison purposes, control cement paste and cement containing pristine materials (Bi_2_O_3_ and Gd_2_O_3_) were produced. As an outcome, synthesis method of radiation shielding additives for cement mortars and concrete applications was proposed. 

## 3. Materials and Methods

### 3.1. Materials

**Figure 1:**
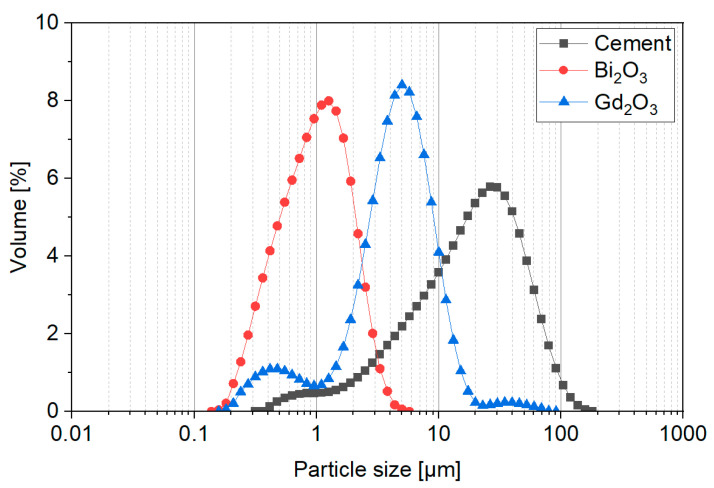
Particle size distribution (by volume) of Bi_2_O_3_, Gd_2_O_3_ and cement. Reprinted from Cendrowski et al. [47].

To produce cement pastes, cement CEM I 42.5 R (Górażdże, Poland) and tap water were used. Commercially available bismuth oxide (Bi_2_O_3_—cat. No. 637017) and gadolinium oxide (Gd_2_O_3_—cat. No. 278513) were purchased from Merck, Poland. The densities of Bi_2_O_3_ and Gd_2_O_3_ determined using a helium pycnometer (Ultrapyc 1200e, Quantachrome Instruments, Anton Paar Group AB, Boynton Beach, FL, USA) were 8.68 g/cm^3^ and 7.28 g/cm^3^, respectively. To produce the silica coatings, tetraethyl orthosilicate (TEOS) was used (Merck, Warsaw, Poland) as a source of silica. Ethylene alcohol (ETOH) and 26% ammonium solution (NH_4_OH) were purchased from Warchem, Poland. Figure 1 presents the particle size distribution determined by the laser diffraction method (Mastersizer2000,Malvern Pananalytical, Malvern, Worcs, UK) of pristine Bi_2_O_3_ and Gd_2_O_3_ particles and cement. Both powders were found to be finer than cement, with the highest fineness of Bi_2_O_3_. The detailed description of primary particles will be discussed in detail in Section 4.1.

### 3.2. Synthesis Process of Functional Additives

The synthesis of silica coatings was performed using two methods marked as A and B. Details on silica synthesis methods were described comprehensively in the authors’ previous work [47] related to silica coating of singular particles. In both methods, equal amounts of the Bi_2_O_3_ and Gd_2_O_3_ were used. For each singular synthesis silica, regardless of method (A or B), a fixed amount of 45 g of Bi_2_O_3_ and Gd_2_O_3_ (mass ratio 1:1) was used.

**Figure 2:**
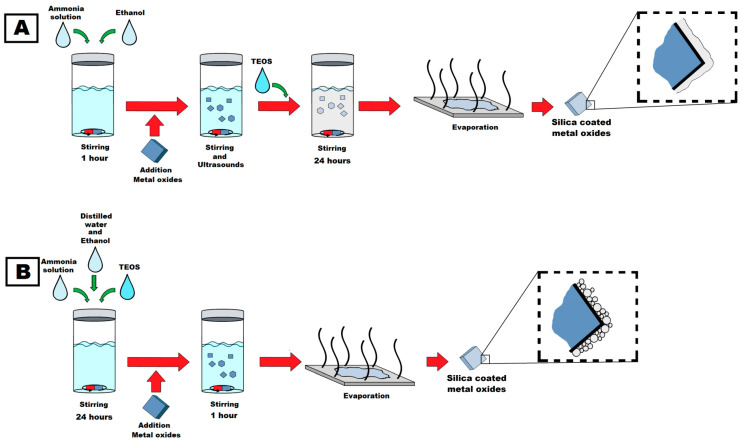
Scheme of the particle coating with the silica according to method A (**A**) and method B (**B**).

In method A (Figure 2A), 900 mL of ethylene alcohol (ETOH) and 45 mL of ammonium solution were mixed using a magnetic stirrer. After 1 h of mixing, the mixture of the Bi_2_O_3_ and Gd_2_O_3_ was dispersed in the prepared solution. To disperse metal oxides, a magnetic stirrer and ultrasound were used alternately. Further to the obtained suspension, 27 mL of TEOS was added into the flask and stirred continuously for 24 h at room temperature. In the ending stage, the suspension was dried in air at 80 °C.

Synthesis of silica type B (Figure 2B) was performed in a mixture of 720 mL of distilled water, 180 mL of ETOH and 45 mL of ammonium solution. A mixture of solvents was poured into the flask and stirred using a magnetic stirrer. Further to the stirred mixture, 27 mL of TEOS was added. Afterward, the solution obtained was stirred for 24 h, with the stirrer rotation speed of 500 rpm. After 24 h, metal oxides were added to the suspension and stirred for an additional hour. In the last step, the suspension was cast on the flat surface of the metal trays and evaporated in air at 80 °C for 24 h.

### 3.3. Mixture Design and Specimen Preparation

To assess the performance of synthesized structures, four representative Portland cement paste (Table 1) mixes were designed with a fixed water-to-binder (w/b) ratio of 0.4. In mixtures containing pristine nanoparticles and synthesized structures, a fixed 1:1 mass ratio of Bi_2_O_3_ to Gd_2_O_3_ was set. In nanomodified mixes, cement was replaced with 10% (by weight) pristine or synthesized structures. Therefore, the content of singular nanoparticles does not exceed a widely agreed state-of-art optimal dosage of 5 wt%. A control cement paste (without additives) specimen was designated as control.

Cement pastes were mixed using an automatic hand mixer. Before mixing, nanoparticles were mixed with tap water (mixing water) and ultrasonicated for 10 min using an ultrasonic bath. The mixing process of cement paste lasted 180 s following the procedure: (i) 30 s slow mixing, (ii) 60 s fast mixing, (iii) 30 s rest, (iv) 60 s fast mixing. After mixing, the material was used to determine its fresh properties, while the rest of the material was used to cast 20 × 20 × 20 mm specimens. After casting, specimens were covered with foil and cured at room temperature for 24 h (T = 20 °C ± 1 °C). Afterward, cubes were demolded and stored in the container up to 28 d with RH = 95% and T = 20 °C ± 1 °C.

### 3.4. Methods

#### 3.4.1. Nanomaterials Characterization

To assess the morphological properties and composition of pristine and synthesized structures, transmission electron microscopy (TEM, Tecnai F30 with a field emission gun operating at 200 kV, Thermo Fisher Scientific, Waltham, MA, USA) with energy dispersive X-ray spectroscopy—EDS (EDX, FEI Corporation, Hillsboro, OR, USA) was used. The crystallinity and phase composition of nanomaterials were characterized using an Aeris diffractometer (Malvern Pananalytical, Malvern, Worcs, UK) with Cu-Kα radiation (λ = 1.544 Å). The BET specific surface area was measured using an N_2_ adsorption/desorption isotherm (Micromeritics ASAP 2460, Norcross, GA, USA). Ultrapyc 1200e (Quantachrome) helium pycnometer was used to assess the specific gravity of newly synthesized structures.

#### 3.4.2. Rheological Properties

Consistency of cement pastes (spread diameter) was determined using a mini-cone that was 36 mm (top diameter) × 60 mm (bottom diameter) × 60 mm (height). A similar mini-cone geometry was used in other studies for cement paste purposes [47,56]. The mini-cone was placed on the flow table and raised vertically. Afterwards, 15 hits of the flow table were applied and the spread diameter was determined in two perpendicular directions, with the mean value taken as a representative.

The rheological properties of cement pastes were measured using an MCR 72 (Anton Paar) compact rheometer with vane geometry. A ribbed cup with volume (v=120 cm^3^) was used to prevent a wall slip effect. In the first stage of the test, the mixture was pre-sheared for 30 s at a constant shear rate of 100 s^−1^ and afterward, the shear stress of mixtures was determined for a total of 300 s at 20 points distributed on a logarithmic scale with shear rates ranging from 100 s^−1^ to 0.1 s^−1^.

Various rheological fitting models such as the linear Bingham model (BM), modified-Bingham model (MBM), and Herschel–Bulkley model (HBM) have been employed to address the non-Newtonian and pseudoplastic behavior of cement-based mixtures [57,58]. In this study, the HBM was found to be the most accurate in fitting the data with a minimum correlation coefficient (R^2^) of 0.99697. The yield shear stress (τ_0_), flow rheology index (n) and consistency coefficient (K) of each mixture were determined using (Equation (1)). In the Herschel–Bulkley model, the consistency coefficient serves as a crucial parameter that gauges a material’s ability to resist flow after surpassing the yield stress. This coefficient is indicative of the material’s internal structure and degree of internal friction, essentially revealing the material’s resistance to deformation. The flow rheology index is another important parameter that characterizes the non-Newtonian behavior of a material. It defines the nature of the material’s flow after exceeding the yield stress. This parameter depicts the shear-thickening and shear-thinning behavior of the material. Shear-thickening occurs when the n>1 and shear-thinning occurs when the n < 1 [59].

(1) $$\tau =\tau_{0}+K\gamma^{n}$$

#### 3.4.3. Isothermal Calorimetry

Hydration of cement paste was monitored using a TAM Air 8-channel calorimeter (TA Instruments, New Castle, DE, USA) at 20 °C for 168 h. For the measurement, the 12.6 g of cement paste was placed in a 20 mL PE vial, tightly sealed with a screw cap and inserted into the calorimeter. The vials with sand were used as a reference.

#### 3.4.4. Thermogravimetric Analysis (TGA)

Thermogravimetric analysis (TGA) of cement pastes after 2 d, 7 d and 28 d of curing was performed using TA Instruments 5500 in nitrogen, with a heat rate 10 °C/min, up to 1000 °C. To perform the measurement, the cubic specimens were crushed and immersed in the solvent (isopropanol) to stop the hydration process for 1 h. Afterward, the material was dried using a vacuum dryer (40 °C) and milled to fine powder. The mass loss caused by the dehydroxylation of calcium hydroxide (CH) in a temperature range of around 400 °C to 500 °C was determined with the tangential method, according to Scrivener et al. [60]. The following equation was used to determine CH content in the paste (Equation (2)):

(2) $$W_{\left[{Ca(OH)}_{2}\right]}=\frac{M_{400{}^{\circ}C-500{}^{\circ}C}}{M_{1000{}^{\circ}C}}\times \frac{m_{\left[{Ca(OH)}_{2}\right]}}{m_{\left[H_{2}O\right]}}$$

where $W_{\left[{Ca(OH)}_{2}\right]}$ is the mass of calcium hydroxide in the paste, $M_{400{}^{\circ}C-500{}^{\circ}C}$ is the range of mass loss caused by the dehydroxylation of calcium hydroxide (determined using the tangential method), $M_{1000{}^{\circ}C}$ mass of specimen at 900 °C and $m_{\left[{Ca(OH)}_{2}\right]}$ and $m_{\left[H_{2}O\right]}$ represent the molar masses of calcium hydroxide (74 g/mol) and water (18 g/mol).

#### 3.4.5. Mechanical Performance

The compressive strength of specimens was determined on 20 mm × 20 mm × 20 mm specimens using electromechanical universal tester UNIFRAME 250 (CONTROLS S.p.A., Liscate, Italy) with a loading rate of 0.6 MPa/s. The measurement was performed after 1 d, 2 d, 7 d and 28 d of curing. Three specimens were tested and the mean value was taken as a representative.

#### 3.4.6. Mercury Intrusion Porosimetry

To analyze the microstructural properties, mercury intrusion porosimetry tests were performed using Quantachrome Poremaster 60. The specimens in question (0.7 cm × 0.7 cm × 2.0 cm) were cut from the middle section of cubic samples. For each composite, two tests were conducted to verify the repeatability of the results. The tests were carried out after 28 days of composite curing. Before the tests, the specimens were dried at 70 °C for 48 hours. The mercury surface tension was set at 0.48 N/m and the contact angle was set to 140° upon intrusion. The specimens were placed into measurement cells and filled with mercury in a low-pressure chamber (to 0.34 MPa). Furthermore, the cells were inserted into a pressure chamber and subjected to high pressures (up to 413 MPa). Based on the tests, cumulative and log differential graphs of porosity distribution were made and the basic properties of composites were given, such as porosity, specific surface area and volume density.

### 3.5. Radiation Shielding Performance Test

In this study, two silica-coated Bi_2_O_3_/Gd_2_O_3_ structures, A and B, were utilized to produce silica nanospheres. These methods yielded distinct structures while preserving the same theoretical elemental composition. It is theoretically unfeasible to determine the expected difference in radiation capacity due to the fabrication method. Therefore, only the experimental results were considered. The radiation shielding properties of the prepared samples were assessed using narrow-beam transmission geometry.

**Figure 3:**
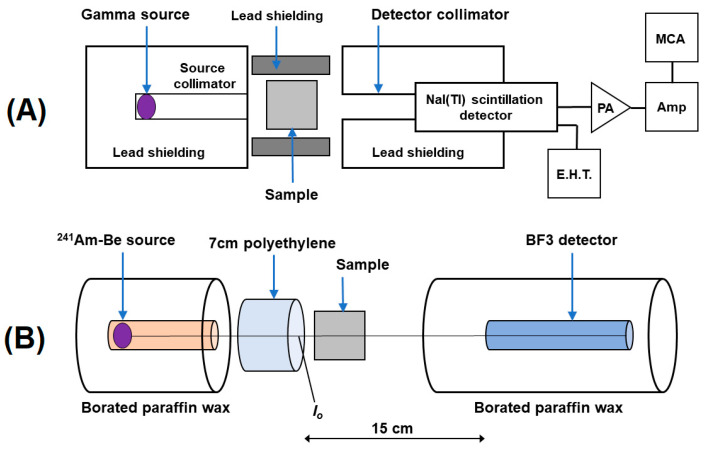
Experimental setup for gamma-ray (**A**) and slow neutron (**B**) transmission. Reproduced from Sikora et al. [8] with permission from Elsevier, 2021.

A comprehensive explanation of the experimental setup was already presented in the previous work of our group [8]. In the gamma-ray experiment, we used gamma-ray lines emitted by radioactive point sources, specifically ^133^Ba, ^137^Cs, ^60^Co and ^233^Th. These sources covered an energy range of 81–2614 keV and were used for both the sample irradiation and detector calibration. To record and analyze the obtained spectra, we utilized a 3″ × 3″ NaI(Tl) scintillator detector, a multichannel analyzer and Genie 2000 software (Canberra). The gamma-ray setup measurements are presented in Figure 3A, which depicts the essential geometry. Furthermore, an investigation on neutrons was performed, utilizing an Am-Be neutron source with an activity of 3.7 GBq. This study was conducted using the experimental setup presented in Figure 3B.

From this geometric configuration and Lambert-Beer law, the following fundamental attenuation coefficients were obtained: the linear coefficient, LAC (μ); mass attenuation coefficient, MAC (μ_m_); total macroscopic cross-section of the slow neutron attenuation coefficient (Σ*_s_*); half-value layer (HVL); and mean free path (MFP) using the equations listed in Table 2.

## 4. Results

### 4.1. Nanomaterials Characterization

**Figure 4:**
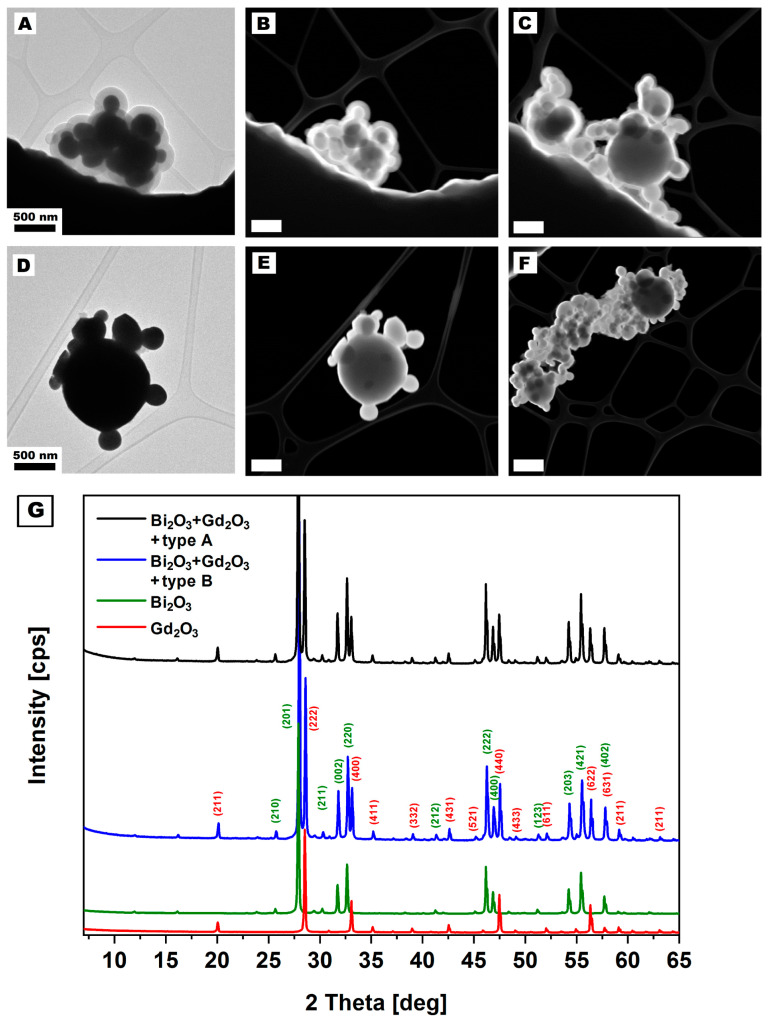
TEM and STEM images of gadolinium and bismuth oxide covered with silica synthesized via methods A (**A**–**C**) and B (**D**–**F**). The white bar on the STEM images correspond to the length of 500 nm. XRD spectrum (**G**) shows signals from the gadolinium and bismuth oxide coated with the silica (via different methods) and pristine metal oxides.

**Figure 5:**
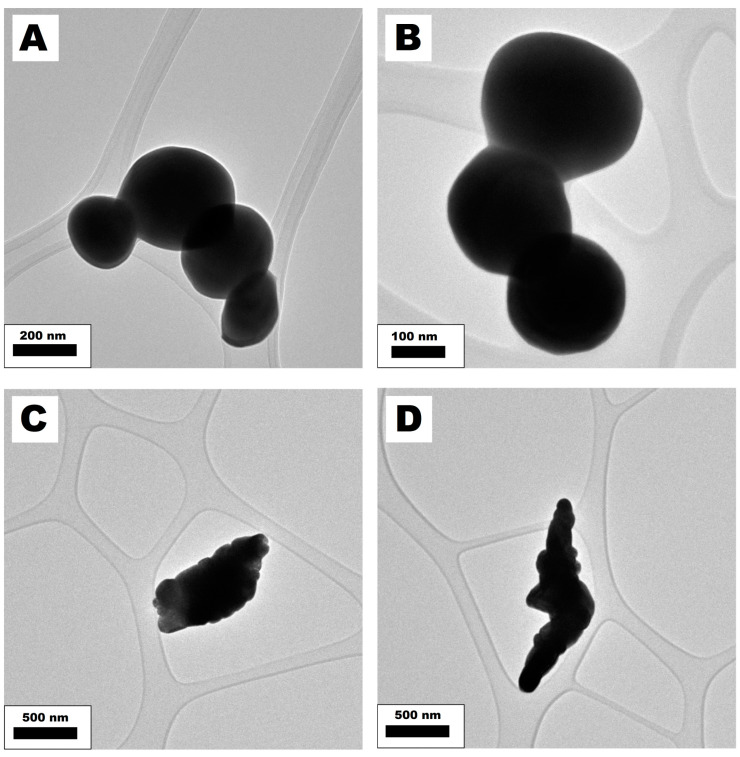
TEM images of pristine Bi_2_O_3_ (**A**,**B**) and Gd_2_O_3_ (**C**,**D**) particles.

The TEM images of gadolinium and bismuth oxide structures after silica coating (Figure 4A,D) show agglomerates made from pristine metal oxide particles. In the agglomerates, two types of particles can be distinguished. Small particles’ diameter ranges from 93 nm to 643 nm, with a mean diameter of about 250 nm corresponding to the size of bismuth oxide particles (according to the TEM analysis, Figure 5A,B). According to the TEM images, bigger and darker particles correspond to the size and shape of pristine gadolinium oxide particles. The Gd_2_O_3_ crystals’ size ranges from 101 nm to 248 nm, with a more spherical and regular shape. The Gd_2_O_3_ crystals are in the form of agglomerates with sizes measured between 256 nm and 2000 nm (Figure 5C,D). The XRD analysis of the bismuth and gadolinium oxides after silica enrichment is presented in Figure 4G. No significant difference between the samples enriched with the silica (type A and B) was noticed. All peaks in the samples were assigned to the β-Bi_2_O_3_ and Gd_2_O_3_ phase, according to the PDF 27-0050 and JCPDS card no. 43-1014, respectively. Additionally, the spectra of pristine metal oxides are presented in Figure 4G. The XRD analyses confirm that the synthesis of silica coatings did not influence the chemical structure of the metal oxide core.

TEM images revealed significant differences between samples enriched with silica according to methods A and B. Silica synthesized employing method A has a solid structure and smooth surface that is free from impurities. The thickness of the silica shell ranges from 26 nm to 35 nm, with a mean thickness of around 30 nm. The TEM image analysis estimated the thickness of the silica shell. High-magnification TEM images of the silica synthesized according to method A are presented in Figure S1A,B (Supplementary Materials) and the previous publication [47]. The introduction of silica coatings resulted in a decrement of the structures’ specific gravities compared to the values reported for singular particles. It is attributed to the substantially lower density of SiO_2_ compared to Bi_2_O_3_ and Gd_2_O_3_ particles (Table 3).

The synthesized silica particles from method B agglomerate at sizes below 15 nm, resembling silica particles (Figure S1 C,D, Supplementary Materials). Such a structure should be characterized by a higher surface area than silica synthesized via method A. Also, in the sample synthesized by method B, additional free silica can be noticed. Other higher magnification TEM images of silica coating over metal oxides are presented in Figure S2 (Supplementary Materials).

The pores and surface area analysis proved the changes in the silica structures. According to BET analysis, Bi_2_O_3_ + Gd_2_O_3_ with silica synthesized using methods A and B have pore volumes of 0.0023 cm^3^/g and 0.04682 cm^3^/g, respectively. Hence, the surface area of the samples reaches 8.0129 m^2^/g and 45.0256 m^2^/g, respectively (Table 3). The difference in the total volume of pores and surface area proves the silica coating’s microscopic analysis.

**Figure 6:**
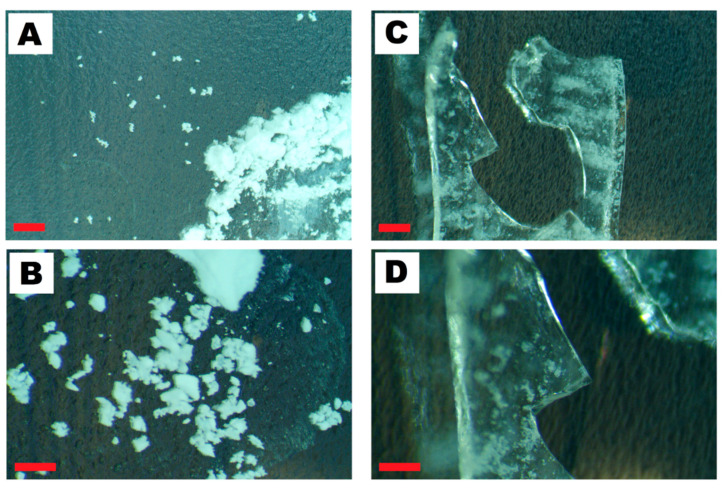
Microscopic images of the silica synthesized via method A (**A**,**B**) and method B (**C,D**). The red scale bar in images (**A**–**D**) represents 1 mm.

Upon comparison of methods A and B, significant differences were observed in the synthesized silica samples under optical microscopic analysis. Silica synthesized via method A has a white color and powder form (Figure 6A,B). This microscopic analysis is in accordance with the previous studies on the synthesis of silica nanomaterials (spheres and coatings) [64,65,66]. These studies indicated that silica synthesized via method A has a non-porous and amorphous character [66].

On the contrary, silica synthesized using method B is transparent and has a crystal form resembling glass. Figure 6C,D shows optical microscope images of dried silica. The translucent pieces of silica can be noticed as white spots. This white powder is micro-sized silica from the crashed, broken or grounded silica pieces. Figure 6D shows the image of the white, small particles at higher magnification. This image proves that white particles have a similar structure/form to the macro-sized silica pieces.

The difference between both methods can also be observed within the synthesized silica particles. The solution from the silica synthesized according to method A changes from transparent and colorless to milky white, whereas the solution remains transparent and colorless during the second method. Our analysis was proven by Yun et al. [67], which shows dependence between the size of the particles and the color of the silica suspensions. As reported, silica up to 30 nm shows a tendency to create transparent solutions. Further increase to the size of colloidal silica changes color to white and milky white. Their studies are in accordance with our present and previous studies on nanosilica.

The most crucial advantage of method B over method A is the requirements and complexity of synthesis. Synthesis of SiO_2_ via method B allows for the reduction of 80% of the ethanol by replacing it with distilled water [47], in contrary to method A [64,65]. It allows for a significant decrease in synthesis costs. For synthesizing silica shells, method B offers a considerable advantage over other methods due to its simple and uncomplicated synthesis procedure described in detail in [47]. The complexity of method A compared to method B lies in the synthesis step where metal oxides are introduced and additional treatment of metal oxides is required. Synthesized silica shell via method A requires proper homogenization of metal oxides using stirring and sonication in the solution before TEOS hydrolyses and condensation. In method B, metal oxides are introduced after TEOS hydrolyses.

### 4.2. Fresh Properties

**Figure 7:**
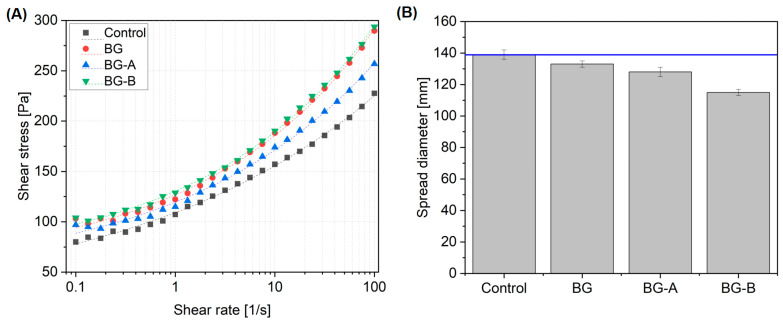
Rheology test results (**A**) and consistency (mean diameter) determined with a mini cone using the flow table method (**B**).

The rheology measurements were performed on all the mixtures, and the principal rheology parameters, including yield shear stress, consistency coefficient and rheological index of the mixtures, were calculated using the HBM fitting model (Figure 7). Table 4 shows that all the mixtures have n < 1, suggesting that the mixtures exhibit shear-thinning behavior.

The results indicated that the incorporation of BG additives without coating results in a substantial increase in the yield shear stress of the mixture. As can be seen in Table 4, the yield shear stress of the paste increased from 15.9 Pa for the control sample and reached 53.2 Pa for the BG mixture. The incorporation of Bi_2_O_3_ and Gd_2_O_3_ nanoparticles in pastes increases yield shear stress. This effect can be attributed to the improved particle packing and interlocking within the paste matrix, which leads to enhanced resistance against deformation and flow. The nanoparticles improve the interparticle forces, resulting in stronger particle–particle interactions and a more compact microstructure. These changes contribute to the increased yield shear stress observed in the pastes.

The coating implementation, however, results in a distinct alternation in the rheological parameters of the fresh mortar. The results indicated that the mixture containing BG particles modified through method A exhibited a reduction in the yield shear stress compared to plain BG additive incorporation, while the consistency coefficient slightly increased. The results indicate that the BG-A showed a decrease in yield shear stress (i.e., about 19%) and a slight increase in consistency coefficient (i.e., 4%) compared to the BG. The coating process could have influenced the dispersion and aggregation behavior of the BG particles. Improved dispersion through method A might have reduced particle aggregation, resulting in a more dispersed state within the paste. Moreover, as confirmed in the TEM results, coating method A produces particles with smooth surfaces free from impurities. Enhanced dispersion coupled with the presence of particles with smooth surfaces decrease the internal friction and facilitate the material flow under shear force, reducing yield shear stress. Moreover, silica coating can act as a lubricant or flow-enhancing additive due to its low friction and smooth surface properties. The presence of silica powder as a coating on BG particles can contribute to reduced friction and resistance to flow, leading to a decrease in yield shear stress.

Coating method B, on the other hand, results in a slight increase in the yield shear stress and consistency coefficient by 6% and 0.5%, respectively, compared to the values registered for the mixture containing plain BG. The reason has to be associated with the increased interaction between the particles within the mixture. The utilization of method B to coat BG particles with silica powder could introduce additional interparticle forces, such as Van der Waals forces or electrostatic interactions. These increased interactions enhance particle interlocking, resulting in higher yield shear stress. At the same time, the improved particle–particle interaction can increase internal friction and resistance to flow, leading to an increase in consistency coefficient.

Figure 7B depicts a mini-slump flow test measuring fresh concrete’s overall consistency or workability. The results indicated that the inclusion of BG particles led to a slight reduction in the spread diameter from 139 mm for the control mix to 133 mm for the BG.

Employing silica coating method A on BG particles resulted in a decrease in spread diameter to 128 mm when compared to the control sample. The highest reduction in spread diameter was registered for the sample coated with method B, with a 14% spread diameter reduction compared to that of the BG mixture. The observation is in line with the previous study [68], confirming that due to large specific surface area of nanosilica the consistency of cementitious composites have a tendency to decrease. The results obtained through mini-slump tests do not correspond with the trends found on dynamic yield shear stress values obtained in the rheology measurements. Previous research [69] has also suggested that basic assessments such as flow table and slump may not accurately read rheological characteristics.

### 4.3. Isothermal Calorimetry

**Figure 8:**
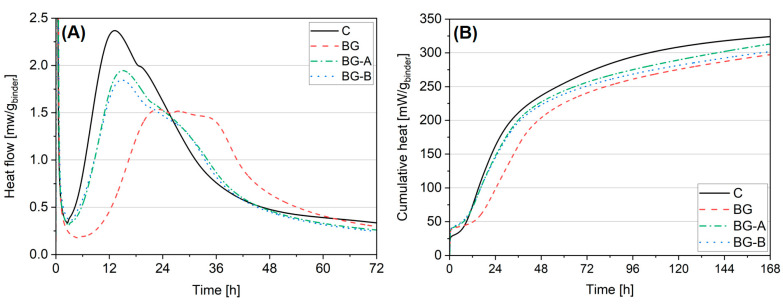
Heat flow (**A**) and cumulative heat (**B**) released from pastes up to 168 h of hydration.

Figure 8 presents a heat flow, and cumulative heat curves of cement pastes tested up to seven days of hydration. In this study, the isothermal calorimetry tests were conducted to investigate the hydration behavior of the mixtures, aiming to identify the reason for the early age compressive strength reduction (Section 4.5). For better readability and discussion, the heat flow results (Figure 8A) were presented up to 72 h.

A remarkable delay in the hydration process of BG paste, when compared to control paste, can be noticed. The occurrence of the second hydration peak in BG was delayed by 71%, and the magnitude of the peak was decreased by 35% when compared to the Control, as shown in Table 5. Similarly, the cumulative heat release of the BG specimen was found to be substantially decreased, especially in the first 48 h of hydration. When compared to the control specimen, the BG paste released only 67%, 55% and 61% percentage of heat emitted by the control paste after 12 h, 16 h and 24 h, respectively. After 168 h, the total heat released from the BG specimen was 8% lower compared to the control paste, which can be attributed to the mass replacement rate of cement with BG. The primary factor responsible for the delayed hydration of cementitious systems containing BG is related to the retarding effect of Bi_2_O_3_ nanoparticles. Cendrowski et al. [47] confirmed that a cementitious system comprising 5 wt% and 10 wt% cement replacement with nanosized Bi_2_O_3_ delayed the occurrence and power of a second exothermic peak from 12 h 54 min (power of 2.37 mW/g^−1^) to 22 h 21 min (power of 1.65 mW/g^−1^) and 29 h 53 min (power of 1.48 mW/g^−1^), respectively.

Contrary to Bi_2_O_3_, available literature suggests that lower dosages of Gd_2_O_3_ have either a neutral or slightly accelerating effect on the cement hydration process. In comparison, higher dosages (up to 10 wt%) could lead to slight retardation of the cement hydration process [47]. Therefore, based on our study, it can be concluded that the primary cause of retardation of the hydration process of BG cement paste is the presence of Bi_2_O_3_ in the cementitious system.

**Figure 9:**
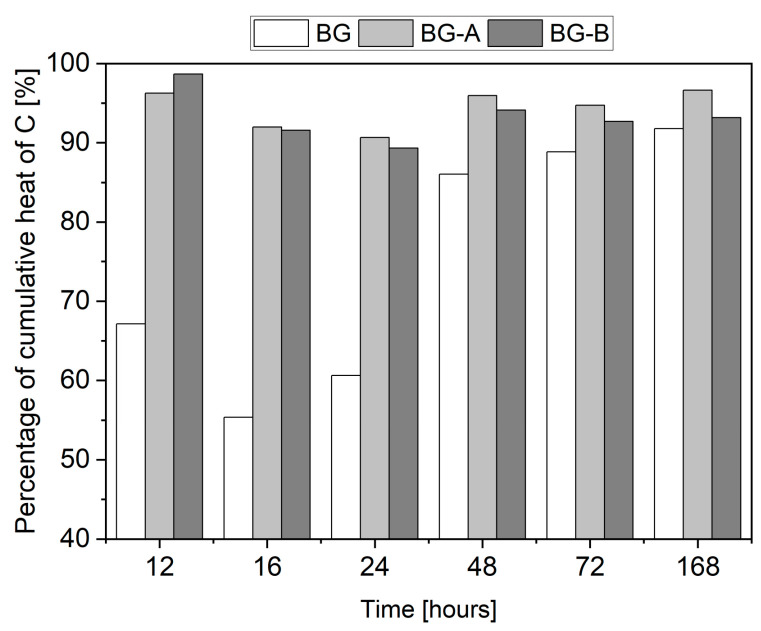
Percentage of cumulative heat released from pastes at different hydration times compared to control paste (C).

The incorporation of silica-coated Bi_2_O_3_-Gd_2_O_3_ structures resulted in a remarkable acceleration of the hydration process of cementitious systems when compared to BG and, thus, a substantial reduction of the negative impact of Bi_2_O_3_ presence in the paste (Figure 9). Compared to the control paste, the occurrence of a second exothermic peak was delayed only by 15% and 14%. In comparison, the peak’s magnitude decreased by 17% and 21% for specimens BG-A and BG-B, respectively (Table 5). Contrary to the BG specimen, pastes containing synthesized silica-coated structures (BG-A and BG-B), over the entire time of testing, exhibited comparable heat emission to that of the control paste. The emitted heat after 168 h was found to be 3% and 6% lower for specimens BG-A and BG-B, respectively.

A positive effect of newly synthesized structures on the hydration process of cementitious systems can be found, and can be attributed to thin silica-layer coating. Due to the extremely high reactivity of silica, a substantial acceleration of the hydration process is obtained along with improved embedment of the material in the cementitious matrix [70,71,72]. Due to the synthesis process and higher amorphicity level of the solid silica shell (type A coating), higher improvement rates were recorded despite a substantially lower surface area of the material compared to a structure containing type B silica coating, which was further confirmed by TG analysis.

### 4.4. Thermogravimetric Analysis (CH Content)

**Figure 10:**
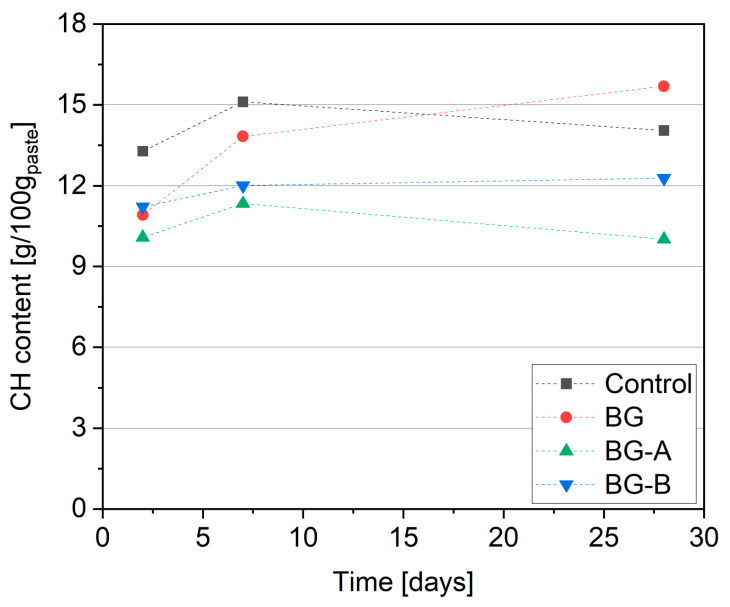
Calcium hydroxide (CH) content in cement pastes determined from TGA.

The results of CH content determined from TG curves are presented in Figure 10. Lower CH content in specimens containing additives can be found after 2 d and 7 d due to a more deficient amount of cement available for hydration when compared to the control paste. The inclusion of synthesized structures resulted in lower CH contents in the BG-A and BG-B pastes as a result of the pozzolanic activity of the silica shell. It is widely agreed that nanosized silica exhibits spectacular pozzolanic activity, and this effect is highly related to various parameters including amorphicity, the size of the particle and its specific surface area [73,74,75,76]. The lowest CH content was reported for BG-A, followed by BG-B, which is in line with the activity of additives in the calorimetric study. After 2 d, the CH content in BG and BG-B specimens was comparable, while BG-A exhibited 24% and 7% lower CH content than control and BG, respectively. After 7 d, BG-A and BG-B showed 18% and 13% lower CH content when compared to BG. After 28 days of curing, the discrepancy between CH content in specimens has been increased, confirming the superior pozzolanic activity of BG-A particles attributed to the highly amorphous level of type A silica coating compared to type B coating. 

### 4.5. Mechanical Performance

**Figure 11:**
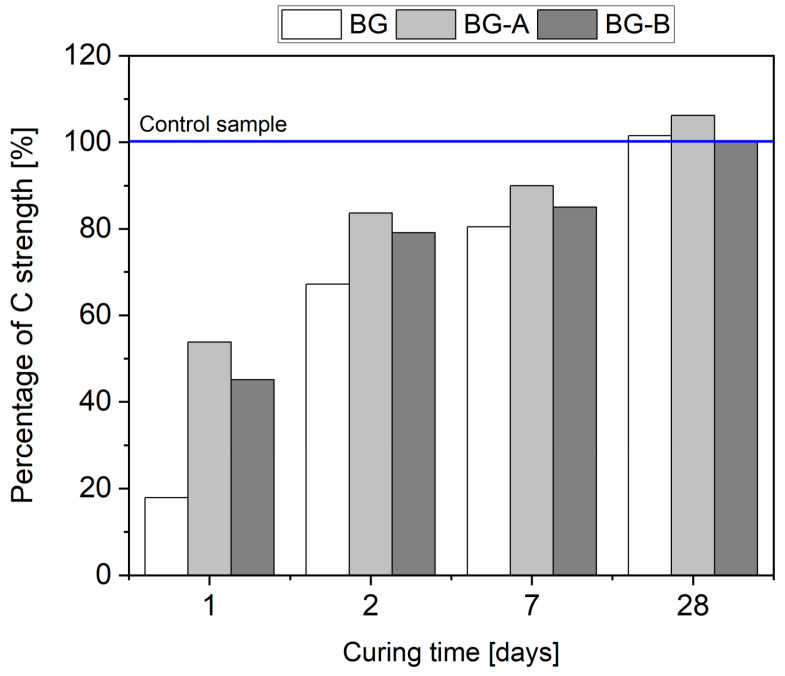
Compressive strength of modified specimens in percentage in relation to the control sample.

Table 6 presents the compressive strength values after 1 d, 2 d, 7 d and 28 days of curing. For more efficient discussion on early age characteristics of the material, compressive strength values were additionally presented as a percentage of the control (C) specimen’s strength at the selected testing date (Figure 11).

As seen in Figure 11, for all the compositions containing BG particles, with and without silica coating, the compressive strength is lower than that of the corresponding control sample at early hydration ages (i.e., 1, 2 and 7 days of curing).

On the first day of the hydration process, paste mixes BG, BG-A and BG-B attained 17.9%, 53.8% and 45.1% compressive strength of control samples, respectively. As the hydration process progressed, the compressive stress of the mixes mentioned above gradually improved and approached that of the control sample. After seven days of curing, the compressive strength of the BG, BG-A and BG-B reached 80.5%, 90% and 93.9% compressive strength of the control samples, respectively. Test results revealed that the silica coating approaches, both methods A and B, exhibited a positive effect, diminishing the delay in the hydration process and resulting in a higher compressive strength at early ages. However, method A showed slightly higher mechanical performance, i.e., 19.2%, 5.7% and 5.9% at 1 d, 2 d and 7 days of curing, respectively, than method B. Several authors [77,78,79] have reported that the impact of various nanoparticle incorporation on the properties of cementitious composites is most pronounced during the early stages of hydration. Nevertheless, acquiring adequate mechanical performance is of utmost importance when considering their practical applications [80]. According to the study’s findings, the inclusion of BG nanoparticles, with or without silica coatings, exhibited no reduction in compressive strength, and all specimens showed comparable value of compressive strength after 28 days of curing despite a decline of cement content in the mix by 10 wt%.

### 4.6. Mercury Intrusion Porosimetry

**Figure 12:**
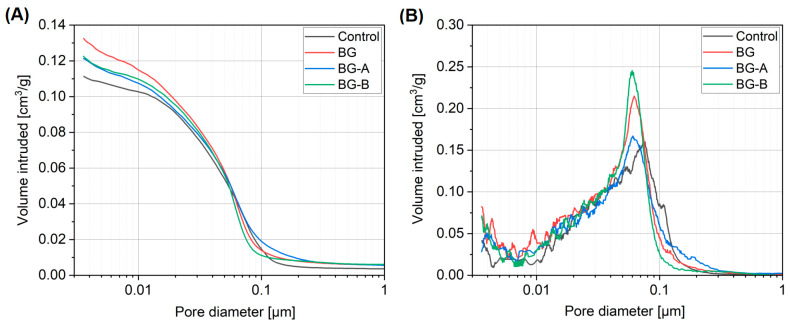
Cumulative (**A**) and log-differential (**B**) pore size distributions of the tested composites.

Paste porosity tests performed using MIP indicate comparable pore distributions in all tested specimens. Comparing the results of porosity distribution (Figure 12) of the tested composites, a slight shift of the pore peak between the control sample and the other pastes can be distinguished (0.075 μm → 0.058 μm). In addition, the BG-B specimen is characterized by the highest value of the pore peak in the range from 0.05 μm to 0.07 μm. In the range from 0.003 μm to 0.02 μm, the pore content in the samples with the addition of metal oxides is clearly higher compared to the control paste, which significantly affected the total pore content and the specific surface area.

Table 7 summarizes the fundamental property values obtained from the MIP measurements. The addition of metal oxides increased the porosity from 1.1% (BG-B) to 2.9% (BG) compared to the control material. It also clearly influenced the value of the specific surface area, which in all cases was higher than in the control sample. The highest surface area was observed in the composite in which the additives were added directly without the silicon coating (BG). The use of a silicon coating in both methods A and B also resulted in an increase in the specific surface area of 21.4% (method A) and 21.8%, respectively (method B). A slightly lower volume density also characterized composites with the addition of oxides compared to the control paste. The obtained results are in line with the theoretical expectations that the replacement of 10 wt% of cement with non-reactive material of higher density will result in higher free water available in the mixture, thus increasing the porosity of the matrix. On the contrary, the inclusion of particles finer than cement resulted in a slight alteration of the pore structure. It shifted towards finer pores in the matrix, resulting in a higher total surface area. In addition, specimens coated with silica resulted in the improvement of the microstructure when compared to BG as a result of higher pozzolanic reactivity of nanosilica, and thus the formation of more refined and compact microstructure, as reported in previous studies [81,82,83].

### 4.7. Radiation Shielding Results

#### 4.7.1. Gamma-Ray Attenuation Performance

**Figure 13:**
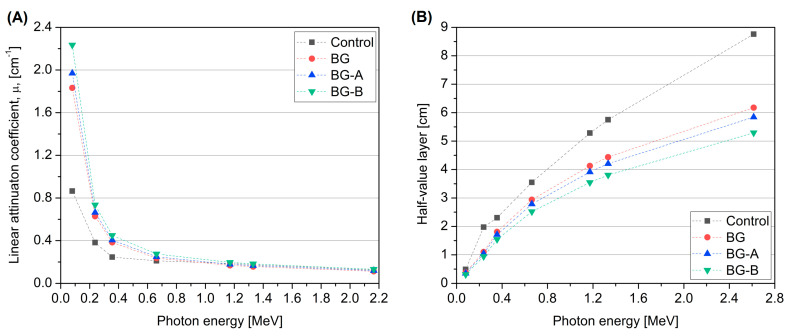
Gamma-ray attenuation parameters of cement paste as a function of photon energy: LAC (**A**) and HVL (**B**).

Figure 13A illustrates the variation in the LAC with photon energy for each composition. As the photon energy increased, gamma-ray attenuation decreased. The LAC is influenced by both the incident photon energy and the metal coating method employed on the specimen. On the other hand, the BG sample, which contains pristine nanoparticles of Bi_2_O_3_ and Gd_2_O_3_, exhibits improved gamma-ray attenuation compared to the control sample. This improvement is due to the highest contributions of Bi and Gd elements.

Figure 13A also showed that the BG-B sample, containing Bi_2_O_3_ and Gd_2_O_3_ coated with silica synthesized using method B, demonstrated higher values compared to the BG-A sample. It can be attributed to nanomaterials’ larger specific surface area, additional free silica, lower total porosity percentage and higher density compared to silica synthesized using method A, as discussed in Section 4.1 and listed in Table 7. As all of these parameters increase the probability of radiation interaction, the BG-B sample performs more efficiently in gamma-ray attenuating than BG-A.

The improvement values listed in Table 8 were calculated for each sample to quantify the increase in attenuation capacity compared to the control sample. According to Table 8, the addition of pristine nanoparticles of Bi_2_O_3_ and Gd_2_O_3_ into BG resulted in a significant increase in shielding performance of about 112% at 80 keV. Additionally, both BG-A and BG-B showed a consistent increase in the linear attenuation coefficient compared to the control and BG samples. Furthermore, BG-B demonstrated an approximately 13.5% improvement in shielding performance compared to BG-A, specifically at 80 keV photon energy. The decrease in improvement values as photon energy increases indicates the increasing dominance of Compton scattering interaction with energy.

The half-value layer (HVL) measures the thickness of the specimen required to reduce the intensity of the incident radiation by half. Therefore, it is useful in assessment of the shielding capacity of any material, particularly for engineering design. Figure 13B displays the HVL data, indicating that the BG-B mix had a lower HVL, which demonstrates its enhanced shielding effectiveness. The gap between the curves in Figure 13 shows a measurable improvement in the shielding capacity.

#### 4.7.2. Slow Neutron Attenuation Performance

Table 9 presents the slow neutron linear attenuation coefficients. The inclusion of pristine Bi_2_O_3_/Gd_2_O_3_ nanoparticles in the cement paste (BG sample) led to notable improvements in the neutron attenuation capacity, which is consistent with similar findings in gamma-ray studies. Table 9 indicates that the cement pastes with Bi_2_O_3_/Gd_2_O_3_ additives exhibited a more significant linear neutron attenuation coefficient (Σ_s_) than the control sample. This increase can be attributed to the contribution of Gadolinium (Gd), which possesses a high thermal neutron capture cross-section and increases the density. Furthermore, Table 9 reveals that the choice of silica-coated Bi_2_O_3_/Gd_2_O_3_ structures resulted in varying levels of improvement in shielding performance. Similar to the gamma-ray studies, silica-coated structure B exhibited a more significant increase in shielding capacity than silica-coated structure A. Using an approach similar to what is shown in Table 8, the improvement values in Table 9 were computed for each mix and compared with the adjacent sample to determine the increase in attenuation capability. Specimens BG, BG-A and BG-B consistently showed higher Σ_s_ values compared to samples BG, BG-A and BG-B. Specifically, method B demonstrated an approximately 34.8% enhancement in slow neutron shielding when compared to coating method A, as indicated in Table 9. Similar enhancements were also observed in terms of HVL and MFP, suggesting an overall increase in slow neutron shielding. Thus, introducing silica-coatings can ultimately enhance the neutron and gamma shielding capabilities, resulting in improved radiation shielding performance.

## 5. Conclusions

Based on the presented research, the following conclusions can be drawn from this work:

1. Two types of silica-coated Bi_2_O_3_/Gd_2_O_3_ structures were synthesized and varied in terms of coating structures, thickness, porosity and surface area, allowing the modification of selected properties such as hydration process, rheology, early strength development and radiation shielding. Proposed structures were found to be beneficial both to early age strength development and radiation shielding properties of cement pastes. Thanks to the introduction of high-Z materials Bi_2_O_3_/Gd_2_O_3_ along with the material of neutron capture cross-section (Gd_2_O_3_), simultaneous improvement of both gamma-ray and neutron attenuation can be achieved.
2. Higher reactivity of synthesized structures, and thus higher acceleration of hydration process and strength development, was obtained in the case of type A silica coating of Bi_2_O_3_/Gd_2_O_3_. Due to the pozzolanic activity of silica coating, lower CH contents were found in specimens containing silica-coated structures when compared to specimens containing uncoated particles. Moreover, higher microstructure refinement in specimens BG-A and BG-B was found compared to specimen BG. Although method B shows slightly lower reactivity in the cement hydration process, it offers advantages related to the additional alteration of rheological properties (increased yield shear stress) which can be useful in selected applications such as 3D printing. At the same time, coating type B has the advantage of lower synthesis time and cost reduction (reduction of ethanol content required for synthesis by 80%).
3. Rheology tests are an essential and effective tool for selecting nanoparticle candidates and determining the most suitable silica coating methods for specific applications. The incorporation of Bi_2_O_3_ and Gd_2_O_3_ particles in pastes increases yield shear stress and consistency coefficient. By employing the silica coating method A on BG particles, the yield shear stress of the resulting pastes decreased by around 19%. On the contrary, the application of method B resulted in a slight increase of 6% compared to the plain BG mixture. These findings highlight the significant impact of silica coating on the properties of BG particles, which can ultimately enhance the performance of resulting composites in the hardened state.
4. Both pristine and silica-coated structures can be effectively used as a cement filler at a rate of 10 wt% without deterioration of 28 days compressive strength. However, due to the extremely retarding effect of the hydration process attributed to pristine Bi_2_O_3_, early age performance of cement pastes containing uncoated structures is deficient. Therefore, the introduction of silica coatings can overcome the retardation of hydration, leading to higher hydration heat and strength gain, especially in the first two days of hydration. Thus, specimens BG-A and BG-B exhibited 300% and 251% (after one day) and 25% and 18% (after two days) higher compressive strength than BG specimens.
5. The incorporation of pristine Bi_2_O_3_ and Gd_2_O_3_ to cement pastes enhanced radiation shielding as expected. The introduction of silica-coated structures resulted in further improvement of the shielding performance of specimens. However, the silica-coated structure synthesized by method B is superior for radiation shielding. The specimens BG-A and BG-B exhibited 7.5% and 13.5% higher LAC, respectively, than the BG specimen at a photon energy of 80 keV. Additionally, the performance of slow neutrons was successfully improved by silica-coated structures. For instance, specimens BG-A and BG-B showed 7.9% and 34.8% higher Σ_s_, respectively, compared to the BG specimen.
6. As an outcome, silica-coated structures can be successfully used in cement-based composites with demanding early age performances, e.g. repairing mortars, prefabrication technology or additive manufacturing (3D printing) where early setting times and high early strengths are crucial factors for these technologies.

## Figures and Tables

**Table 1:** The ratio of the cement paste components (wt%).

Mix	Cement	Water	Bi_2_O_3_	Gd_2_O_3_	Bi_2_O_3_/Gd_2_O_3_-SiO_2_-Type A	Bi_2_O_3_/Gd_2_O_3_-SiO_2_-Type B
Control	1	0.4	-	-	-	-
BG	0.9	0.4	0.05	0.05	-	-
BG-A	0.9	0.4	-	-	0.1 ^1^	-
BG-B	0.9	0.4	-	-	-	0.1 ^1^

^1^ Fixed Bi_2_O_3_ to Gd_2_O_3_ mass ratio of 1:1.

**Table 2:** Equations used to determine gamma-ray and neutron shielding parameters [61,62,63].

Parameter	Symbol	Unit	Equation	Explanation
Linear attenuation coefficient (LAC)	μ	cm^−1^	$\mu =-\frac{1}{x}\ln(I/I_{0})$	x: sample thickness. I_0_ (incident) and I (transmitted) photon intensities.
Mass attenuation coefficient (MAC)	$\mu_{m}$	cm^2^/g	µ_m_ = µ/ρ	ρ: sample density
Macroscopic Slow neutron cross-section	$\Sigma_{s}$	cm^2^/g	$\Sigma_{s}=-\frac{1}{x}\ln(\phi /\phi_{0})$	$\phi_{0}$ (incident) and ϕ (transmitted) neutron fluxes
Half-value layer	HVL	cm	HVL = ln2/µ ln2/Σ*_s_*	-
Mean free path	MFP	cm	MFP = 1/µ; 1/Σ*_s_*	-

**Table 3:** Specific gravity and properties of synthesized structures determined from BET.

Sample	Specific Gravity [g/cm^3^]	BET Surface Area [m^2^/g]	Median Pore Width [Å]	DFT Total Volume in Pores [cm^3^/g]
Bi_2_O_3_	8.68	1.1007	9.116	0.00042
Gd_2_O_3_	7.28	0.1666	7.551	0.00007
Bi_2_O_3_/Gd_2_O_3_/SiO_2_—A	5.46	8.0129	7.652	0.00230
Bi_2_O_3_/Gd_2_O_3_/SiO_2_—B	5.35	45.0256	10.180	0.04682

**Table 4:** Rheological parameters of cement pastes.

Sample Designation	Yield Shear Stress τ_0_ (Pa)	Consistency Coefficient K (Pa·s^n^)	Rheological Index n	R^2^
C	15.9	93.3	0.17	0.99796
BG	53.2	73.7	0.25	0.99759
BG-A	42.9	76.6	0.22	0.99697
BG-B	56.4	74.1	0.26	0.99867

**Table 5:** Maximum exothermic heat rate and peak occurrence.

Mix	Maximum Heat [mW/g]	Loss in Comparison to C [%]	Peak Occurrence [h]	Loss in Comparison to C [%]
Control	2.37	-	12 h 54 min	-
BG	1.53	−35%	22 h 02 min	−71%
BG-A	1.95	−17%	14 h 52 min	−15%
BG-B	1.85	−21%	14 h 44 min	−14%

**Table 6:** Compressive strength evolution of cement pastes.

Mix	1 Day		2 Days		7 Days		28 Days	
	F_c_	SD	F_c_	SD	F_c_	SD	F_c_	SD
Control	17.3	0.60	35.0	2.73	54.7	0.97	58.1	1.91
BG	3.1	0.11	23.5	1.07	44.0	1.83	59.0	0.58
BG-A	9.3	0.47	29,3	0.56	49.2	1.95	61.7	1.15
BG-B	7.8	0.43	27.7	0.51	46.5	0.97	58.2	2.45

**Table 7:** The basic properties of the composited acquired from MIP.

Mix	Total Porosity [%]	Total Surface Area [m^2^/g]
Control	19.74	16.85
BG	22.63	25.61
BG-A	21.15	21.41
BG-B	20.87	21.75

**Table 8:** Experimental LAC for specimens’ control, BG, BG-A and BG-B. LAC improvement factor IMF% of specimens BG, BG-A and BG-B relative to those of samples control, BG, BG-A and BG-B, respectively. Experimental uncertainty ≤ ±3%.

Energy (MeV)	μ (cm^−1^)						
	Control	BG	IMF% ^a^	BG-A	IMF % ^b^	BG-B	IMF% ^c^
0.080	0.8645	1.8325	112.0	1.9693	7.5	2.2344	13.5
0.238	0.38175	0.6279	64.5	0.6634	5.7	0.7348	10.8
0.356	0.245	0.3835	56.5	0.4053	5.7	0.4489	10.8
0.662	0.21075	0.2358	11.9	0.2489	5.5	0.2753	10.6
1.173	0.1679	0.1679	5.5	0.1771	5.5	0.1955	5.0
1.325	0.15626	0.1563	5.5	0.1648	5.5	0.1822	4.6
2.614	0.11222	0.1122	5.7	0.1186	5.7	0.1310	5.1

IMF%a =100×(μBG−μcontrol)/μcontrol, IMF%b =100×(μBG−A−μBG)/μBG, and IMF%c =100×(μBG−B−μBG−A)/μBG−A.

**Table 9:** Experimental slow neutron linear attenuation of prepared specimens. Experimental uncertainty ≤ ±3%.

Parameter	Control	BG	IMF% ^a^	BG-A	IMF% ^b^	BG-B	IMF% ^c^
**Σ_s_ (cm^−1^)**	0.0722	0.0760	5.2	0.0820	7.9	0.1024	34.8
**HVL (cm)**	9.60	9.12	−4.9	8.45	−7.4	6.77	−25.8
**MFP (cm)**	13.85	13.16	−4.9	12.19	−7.4	9.76	−25.8

IMF%a =100×(ΣsBG−Σscontrol)/Σscontrol, IMF%b =100×(ΣsBG−A−ΣsBG)/ΣsBG, and IMF%c =100×(ΣsBG−B−ΣsBG−A)/ΣsBG−A.

## Data Availability

The datasets generated during and/or analyzed during the current study are available from the corresponding author upon reasonable request.

## Supplementary Materials

The following supporting information can be downloaded at https://www.mdpi.com/article/10.3390/nano14020168/s1, Figure S1: TEM images of silica synthesized via method A (images A–B) and B (images C–D); Figure S2: TEM images of silica shell around gadolinium and bismuth oxide covered with silica synthesized via method B.
