# Supplementary material for: Functional Bi2O3/Gd2O3 Silica-Coated Structures for Improvement of Early Age and Radiation Shielding Performance of Cement Pastes
Source: Nanomaterials (Basel). 2024 Jan 12;14(2):168. doi: 10.3390/nano14020168 (PMC10819170; doi:10.3390/nano14020168)
Supplement: Supplementary file 1 [file nanomaterials-14-00168-s001.zip › nanomaterials-2766176-supplementary.pdf]

Functional Bi_2O_3/Gd_2O_3 silica-coated structures for improvement of early age and radiation shielding performance of cement pastes

Krzysztof Cendrowski [1], Karol Federowicz [1], Mateusz Techman [1], Mehdi Chougan [2], Ahmed M. El-Khayatt[3,4], H.A. Saudi [5], Tomasz Kędzierski [6], Ewa Mijowska [6], Jarosław Strzałkowski [1], Daniel Sibera [1], Mohamed Abd Elrahman [7] and Pawel Sikora [1,*]

1 Faculty of Civil and Environmental Engineering, West Pomeranian University of Technology in Szczecin, Poland;
2 Department of Civil and Environmental Engineering, Brunel University London, Uxbridge, UB8 3PH, United Kingdom;
3 Department of Physics, College of Science, Imam Mohammad Ibn Saud Islamic University, (IMSIU), Riyadh, Saudi Arabia;
4 Reactor Physics Department, Nuclear Research Centre, Atomic Energy Authority, 13759, Cairo, Egypt
5 Department of Physics, Faculty of Science, Al-Azhar University, Women Branch, Nasr City, Cairo, Egypt; ;
6 Department of Nanomaterials Physicochemistry, Faculty of Chemical Technology and Engineering, West Pomeranian University of Technology in Szczecin;
7 Structural Engineering Department, Mansoura University, Mansoura City 35516, Egypt;
* Correspondence: pawel.sikora@zut.edu.pl

SUPPLEMENTARY MATERIALS

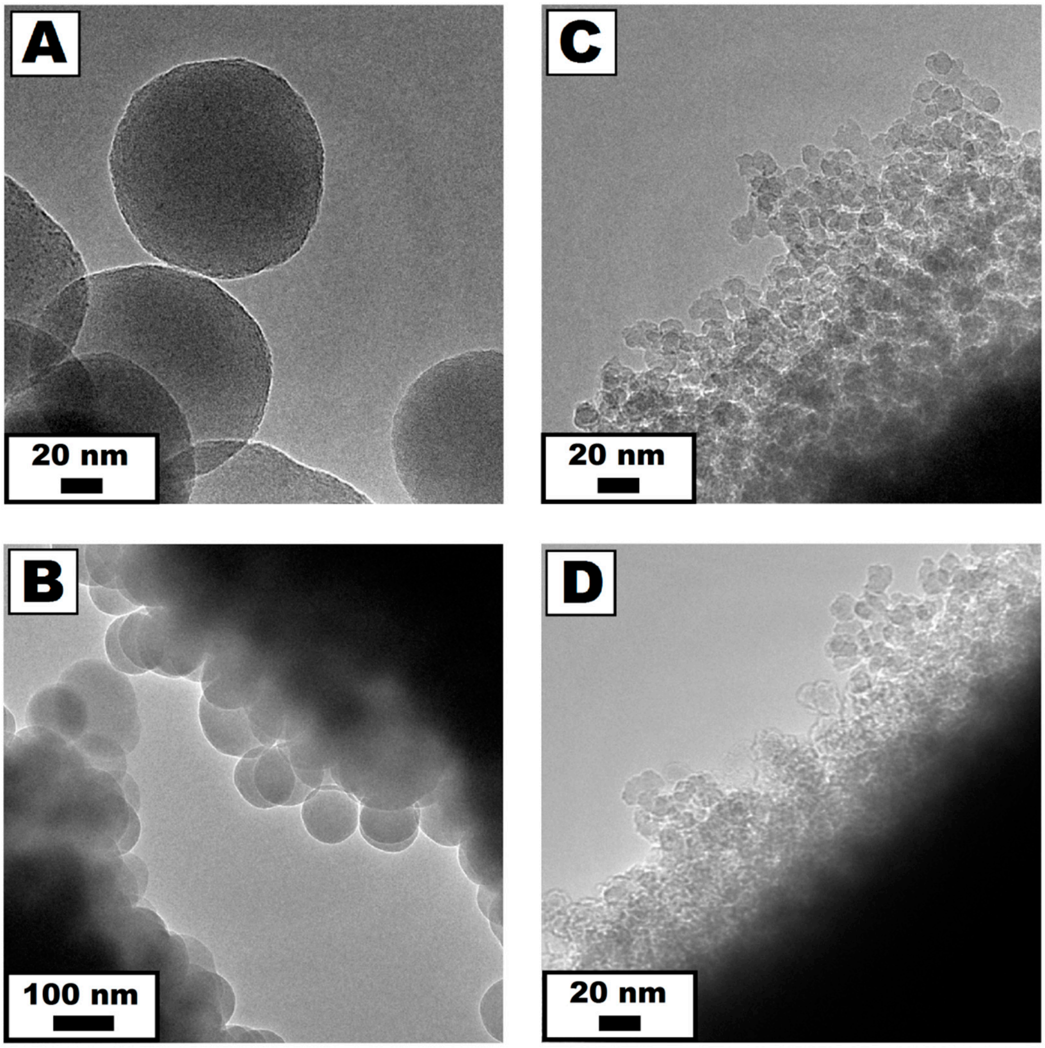

Figure S1. TEM images of silica synthesized via method A (images A - B) and B (images C - D).

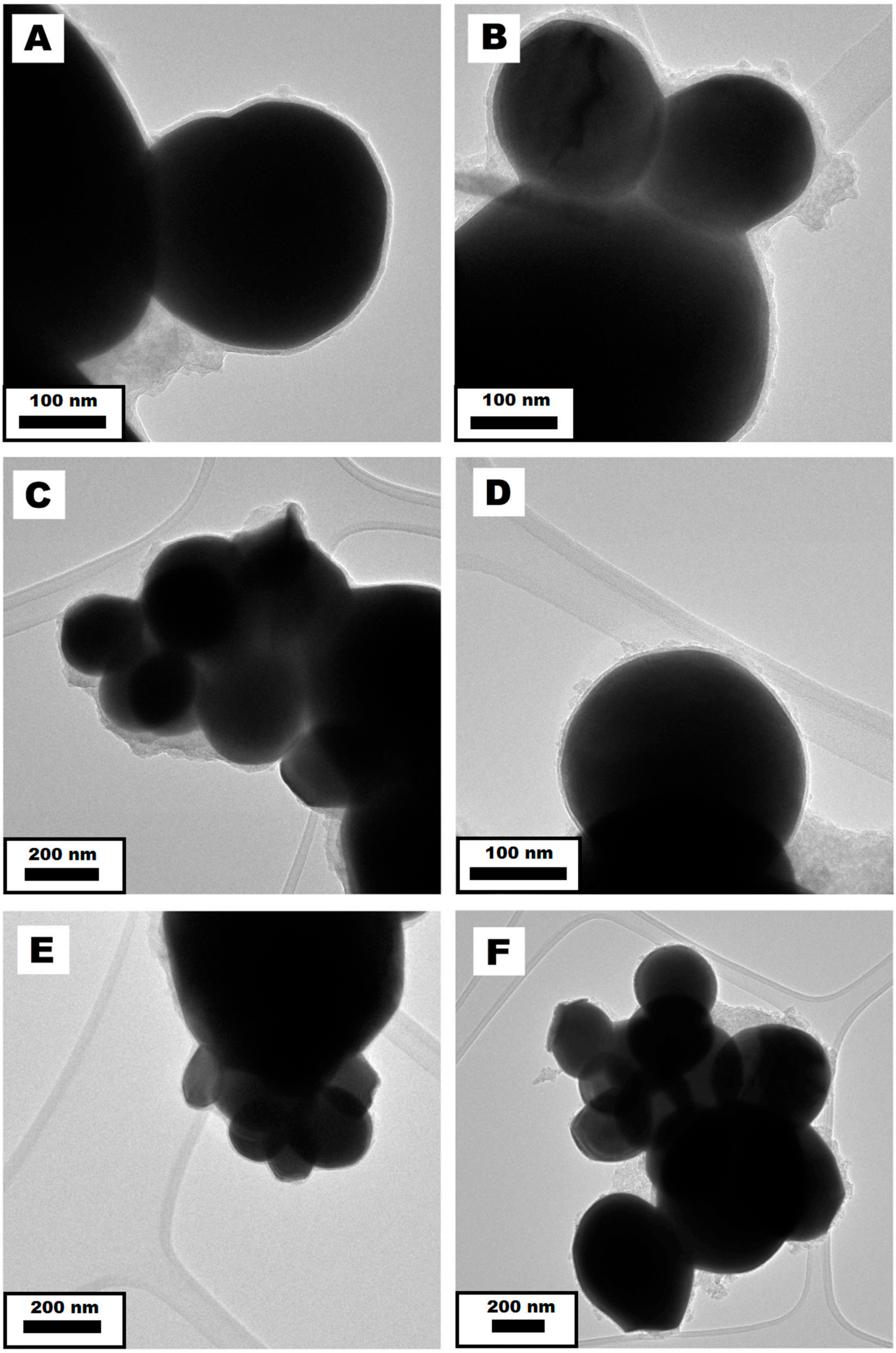

Figure S2. TEM images of silica shell around gadolinium and bismuth oxide covered with silica synthesized via method B.
